# Detection of selenoprotein transcriptome in chondrocytes of patients with Kashin–Beck disease

**DOI:** 10.3389/fcell.2023.1083904

**Published:** 2023-02-17

**Authors:** Yi Gong, Yifan Wu, Yanli Liu, Sijie Chen, Feiyu Zhang, Feihong Chen, Chaowei Wang, Shujin Li, Minhan Hu, Ruitian Huang, Ke Xu, Xi Wang, Lei Yang, Yujie Ning, Cheng Li, Rong Zhou, Xiong Guo

**Affiliations:** ^1^ Department of Occupational and Environmental Health, School of Public Health, Xi’an Jiaotong University Health Science Center, Key Laboratory of Trace Elements and Endemic Diseases, National Health and Family Planning Commission, Xi’an, Shaanxi, China; ^2^ Department of Joint Surgery, Hong Hui Hospital, Xi’an Jiaotong University, Xi’an, China; ^3^ Department of Nursing, Xi’an Jiaotong University, Xi’an, Shaanxi, China; ^4^ Shaanxi Provincial Institute for Endemic Disease Control, Xi’an, Shaanxi, China; ^5^ Clinical Research Center for Endemic Disease of Shaanxi Province, The Second Affiliated Hospital of Xi’an Jiaotong University, Xi’an, Shaanxi, China

**Keywords:** selenoprotein, chondrocytes, Kashin–Beck disease, real-time quantitative polymerase chain reaction, immunohistochemistry

## Abstract

**Background:** Kashin–Beck disease (KBD) is a deformed osteochondral disease with a chronic progression that is restrictively distributed in eastern Siberia, North Korea, and some areas of China, and selenium deficiency has been identified as an important factor in the pathogenesis of this disease in recent years.

**Objective:** The aim of this study is to investigate the selenoprotein transcriptome in chondrocytes and define the contribution of selenoprotein to KBD pathogenesis.

**Methods:** Three cartilage samples were collected from the lateral tibial plateau of adult KBD patients and normal controls paired by age and sex for real-time quantitative polymerase chain reaction (RT-qPCR) to detect the mRNA expression of 25 selenoprotein genes in chondrocytes. Six other samples were collected from adult KBD patients and normal controls. In addition, immunohistochemistry was used on four adolescent KBD samples and seven normal controls (IHC) to determine the expression of proteins screened by RT-qPCR results that had different gene levels.

**Results:** Increased mRNA expression of GPX1 and GPX3 was observed in chondrocytes, and stronger positive staining was displayed in the cartilage from both adult and adolescent patients. The mRNA levels of DIO1, DIO2, and DIO3 were increased in KBD chondrocytes; however, the percentage of positive staining decreased in the KBD cartilage of adults.

**Conclusion:** The selenoprotein transcriptome, mainly the glutathione peroxidase (GPX) and deiodinase (DIO) families were altered in KBD and might play a vital role in the pathogenesis of KBD.

## Introduction

Kashin–Beck disease (KBD) is an endemic, chronic, and deforming osteochondral disease that is characterized by finger enlargement, brachydactyly, joint deformation, and even dwarfism in severe cases (Guo et al., 2014). According to the 2020 China Health Statistics Yearbook (www.nhc.gov.cn), KBD affects 379 endemic districts and counties, with over 103 million residents at risk from northeast to southwest China. A strong hypothesis for the cause of KBD is selenium (Se) deficiency (Kang et al., 2020); however, the underlying mechanisms of the pathological changes in chondrocytes such as necrosis, apoptosis, and extracellular matrix degradation that may be caused by Se deficiency have not been determined, and this information will be vital for formulating prevention and treatment measures for KBD.

Se is one of the most essential micronutrients and has a close relationship with human health and various diseases, such as male infertility, coronary heart disease, seizures, and different kinds of cancer (Rayman, 2012). The physiological function of Se is considered to be in the form of the amino acid selenocysteine (SEC). SEC is bound to the amino acid sequence of selenoproteins during translation and is encoded by the UGA in the coding region of mRNA. SEC tRNA, encoded by Trsp, is responsible for the expression of all selenoproteins by recognizing UGA. Targeted deletion of Trsp in skeletal precursor cells led to impaired growth and development (Downey et al., 2009). Twenty-five selenoproteins have been isolated in humans, and they have been categorized into glutathione peroxidases (GPXs), thioredoxin reductases (TrxRs), and deiodinases (DIOs) based on their functions (Chu et al., 2004). For example, GPX1 is a well-known antioxidant enzyme that can effectively eliminate the harmful accumulation of hydrogen peroxide in cells by reacting with hydrogen peroxide and soluble low-molecular-weight hydrogen peroxide and hydroxide, converting glutathione (GSH) to oxidized glutathione (GSSG) (Margis et al., 2008; Yan et al., 2017). TrxRs cooperate with NADPH and thioredoxin to form the thioredoxin (Trx) system, which provides electrons to thiol-dependent peroxidase to remove reactive oxygen and nitrogen species at a rapid rate (Lu and Holmgren, 2014).

Previous studies suggested lower selenium levels in the plasma and serum of patients with KBD, osteoarthritis (OA), and rheumatoid arthritis (RA) (Yu et al., 2016; Wang et al., 2020; Fu et al., 2022), which implies that Se could play a key role in articular cartilage development and homeostasis. In particular, a meta-analysis reported that the level of serum Se in patients with KBD was significantly lower than that in healthy subjects in all 23 included studies (Yang et al., 2016). Se deficiency not only affects the expression of selenoproteins but also increases the level of intracellular ROS and activates the MAPK signaling pathway (Wang et al., 2022). Wu et al. also reported massive glycogen deposits in KBD chondrocytes and a significantly elevated ROS level, compared to that in normal cells, which suggests that the disorder of glucose metabolism in KBD is involved in the process of chondrocyte injury (Wu et al., 2014a; Wu et al., 2014b).

Moreover, Se supplementation can protect chondrocytes from oxidative damage (Dai et al., 2016) and even reduce the incidence and degree of chondrocyte necrosis in the growth plate of rats caused by a KBD diet (Yang et al., 2017). It also has effective rates of metaphyseal X-ray improvement in intervening trials (Xie et al., 2018). The aforementioned studies suggested that selenium and selenoproteins might play a crucial role in the pathogenesis of KBD. However, few studies have specifically detected the selenoprotein transcriptome in KBD chondrocytes which determines mRNA expression and may affect selenoprotein expression and further alter its biological function directly. At the same time, how these genes are involved in the process of chondrocyte injury in KBD and its mechanisms are still unclear.

In this study, we detected the mRNA expression of 25 selenoprotein genes using real-time quantitative PCR to explore how selenoproteins are involved in the pathogenesis of KBD. Immunohistochemistry (IHC) was used to verify the expression of several selenoproteins (GPX1, GPX3, DIO1, DIO2, and DIO3) in the cartilage tissues from KBD patients and healthy controls.

## Materials and methods

### Disease diagnosis and sample selection

The KBD patients were accurately diagnosed following the national diagnostic standard for KBD in China (WS/T 207–2010). Normal subjects and KBD subjects were selected from the same regions of China. The subjects were matched for age, location, and gender and were all of Han Chinese ethnicity. All subjects were recruited at random from Yongshou County in Shaanxi Province, a region of China where KBD is endemic and has a prevalence of 20.4%. Subjects with additional kinds of osteoarthropathy and other conditions, such as hypertension, coronary heart disease, diabetes, etc., were excluded. In accordance with the criteria for inclusion and exclusion listed previously, adult articular cartilage samples were obtained from three adult individuals with KBD and three normal subjects for RT-qPCR and immunohistochemistry (Supplementary Table S1). The donors of both the adult KBD samples and the control samples were all from the KBD endemic region of Yongshou County. The articular cartilage samples of the adult KBD patients and normal controls were taken from subjects who had undergone knee arthroplasty or had suffered accident-related amputation. Meanwhile, samples of adolescent articular cartilage from the proximal interphalangeal joints of the fingers of four teenage KBD individuals and six normal teenagers were obtained after death for immunohistochemistry (Supplementary Table S1). Accidents or illnesses, including severe diarrhea and acute pneumonia, were the reasons for fatalities. This work has received approval from the Xi’an Jiaotong University Ethics Committee (No. 2022-685).

### Cartilage tissue collection and chondrocyte isolation

Within an hour after surgery, the collection of all samples of the articular cartilage from the lateral tibial plateau, including subchondral bones and all cartilage zones (including calcified tissue), was completed. Articular cartilage specimens were separated into 1 mm^3^ fragments after being washed twice with sterile phosphate buffer containing antibiotics (penicillin and streptomycin). Fragments were digested with 0.25% trypsin for 30 min in an atmosphere of 5% carbon dioxide at 37°C. The cell suspension was transferred into a culture bottle, 0.2% type II collagenase was added, and the cells were digested on a shaker Eppendorf Thermomixer at 37°C for 10 h. Then, the liquid was filtered through 70 mM nylon filters, and the isolated chondrocyte precipitate was obtained by 1000 × g centrifugation (Zheng et al., 2013; Wu et al., 2014a; Wu et al., 2014b).

### RT-qPCR analysis

The mRNA expression levels of 25 selenoproteins were confirmed by RT-qPCR. The total RNA was isolated from chondrocytes by the TRIzol protocol. A RevertAid ™ First Strand cDNA Synthesis Kit (Thermo Scientific Molecular Biology, Canada) was used according to the manufacturer’s instructions to convert RNA into complementary DNA (cDNA), and then, qRT-PCR was performed using an ABI7500 Real-Time PCR system (Applied Biosystems, Foster City, CA, United States). All primers (Supplementary Table S2) and probe sets were supplied in the TaqMan® Gene Expression Assay kits (Applied Biosystems). The relative gene expression levels of selenoproteins in both cases and controls were identified by the 2-△△C(t) method. GAPDH was used as an internal control to normalize the sample differences.

### Immunohistochemistry

The cartilage tissue was immediately fixed in 4% (w/w) paraformaldehyde for 24 h and then transferred to 10% (w/w) disodium ethylenediamine tetraacetate (EDTA-Na_2_) to decalcify for 2 to 3 weeks. The samples were dehydrated with gradient concentration alcohol, cleared with xylene, and finally embedded in paraffin wax. Then, the embedded paraffin samples were cut into 5-micron slices, affixed to slides, and stored at room temperature. Before dyeing, the slices were baked at 65°C for 1 h, dewaxed with xylene, and then rehydrated under the condition of reducing the concentration of ethanol. A proper amount of 0.1% trypsin was added to cover the tissue wax for antigen repair, and it was incubated at 37°C for 30 min and washed three times with 1 × PBS. The endogenous peroxidase activity was blocked with 1.5% (w/w) hydrogen peroxide at room temperature for 10 min and then the slices were washed with 1 × PBS. A 5% normal goat serum working solution was added to the tissue wax block and sealed for 15 min at room temperature. Then, anti-GPX1/anti-GPX3/anti-DIO1 (1:50 dilution ratio, bs-11790-1-ap, protein technology)/anti-DIO2 (1:100 dilution ratio, bs-3673R, Bioss)/anti-DIO3 (1:50 dilution ratio, bs-40229) were added to the area, and the tissue wax block was kept at 4°C overnight (and immunoglobulin G was used as a negative control). The slices were washed with 1 × PBS and incubated using the rabbit SP kit (rabbit streptomycin-biotin detection system) (SP-9001, Zhongshan Jinqiao, Guangzhou, China) according to the manufacturer’s instructions. Freshly prepared DAB chromogenic solution (ZLI-9018, Zhongshan Jinqiao, Guangzhou, China) was added for slice staining and rinsed off gently with tap water. Hematoxylin re-staining, hydrochloric acid alcohol differentiation, flushing, and ammonia anti-blue addition were all carried out. Finally, the slices are dehydrated and installed under an alcohol-washed envelope. The two pathologists carried out and interpreted the IHC staining results under an optical microscope without knowing the source of the sample. The three-layer zones of cartilage joints were determined, according to the different morphologies of the cells, and three or more visual fields were randomly selected for statistical analysis of the positive staining rate (Schumacher et al., 1994; Lorenzo et al., 1998; Karlsson and Lindahl, 2009). In detail, the long axis of the superficial chondrocytes is parallel to the cartilage surface, and the cells are smaller and flatter than those in the middle and deep zones. The middle zone is randomly distributed in the matrix, and the deep zone is perpendicular to the surface, in which the cells have obvious columnar arrangement characteristics. We chose five visual fields of the same size randomly for each zone and counted at a magnification of 50.

### Statistical method

The experimental data were statistically analyzed by the SPSS 18.0 package. After the normality test for continuous variables, when it was satisfied with the normal distribution, one-way analysis of variance (ANOVA) was used to compare the differences between the means, and Tukey’s post hoc test was used for multiple comparison studies; a Student’s t-test was used to evaluate the difference between two groups. If it was not normally distributed, non-parametric methods would be used. A *p*-value < 0.05 was considered to indicate a significant difference. Some experimental results are presented as bars, drawn using GraphPad PRISM 6 (GraphPad, San Diego, CA, United States).

## Results

### Expression levels of the selenoprotein gene mRNA in chondrocytes from KBD patients

Articular cartilage samples from three adult patients with KBD and three normal controls were collected. The total RNA was extracted and converted into cDNA, and the gene expression of all 25 selenoproteins was measured by qRT-PCR.

**FIGURE 1:**
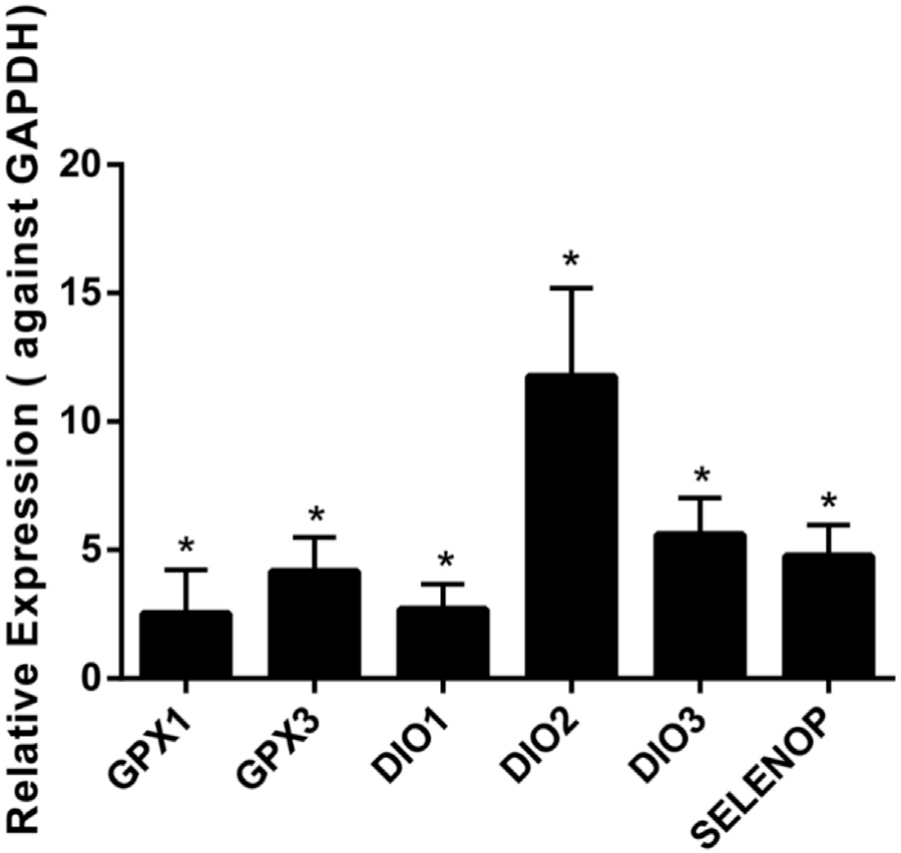
Differentially expressed gene expression of selenoproteins in chondrocytes of KBD patients (*n* = 3) and normal controls (*n* = 3) using qRT-PCR. *p < 0.05.

The results demonstrated that the levels of six selenoprotein genes (GPX1, GPX3, DIO1, DIO2, DIO3, and SELENOP) were upregulated significantly in chondrocytes from KBD patients compared to chondrocytes from normal subjects (Figure 1). The mRNA levels of GPX1 and GPX3 were 2.5 times and 4.2 times higher, respectively, in the chondrocytes of KBD patients than in normal chondrocytes. Additionally, the mRNA levels of DIO1, DIO2, and DIO3 were all increased in KBD chondrocytes compared to normal chondrocytes (a 2.7-fold increase in DIO1, a 11.8-fold increase in DIO2, and a 5.1-fold increase in DIO3). It is also worth noting that the mRNA expression level of SELENOP was 4.8 times higher in KBD chondrocytes than in normal chondrocytes. The mRNA expression levels of the GPX2, GPX6, and SELENOV genes were not detected in chondrocytes in this experiment; however, there was no significant change in the mRNA expression levels of the other selenoprotein genes examined in this study (Supplementary Figures S1, S2).

### IHC verification of differential selenoprotein expression in KBD cartilage tissues

**FIGURE 2:**
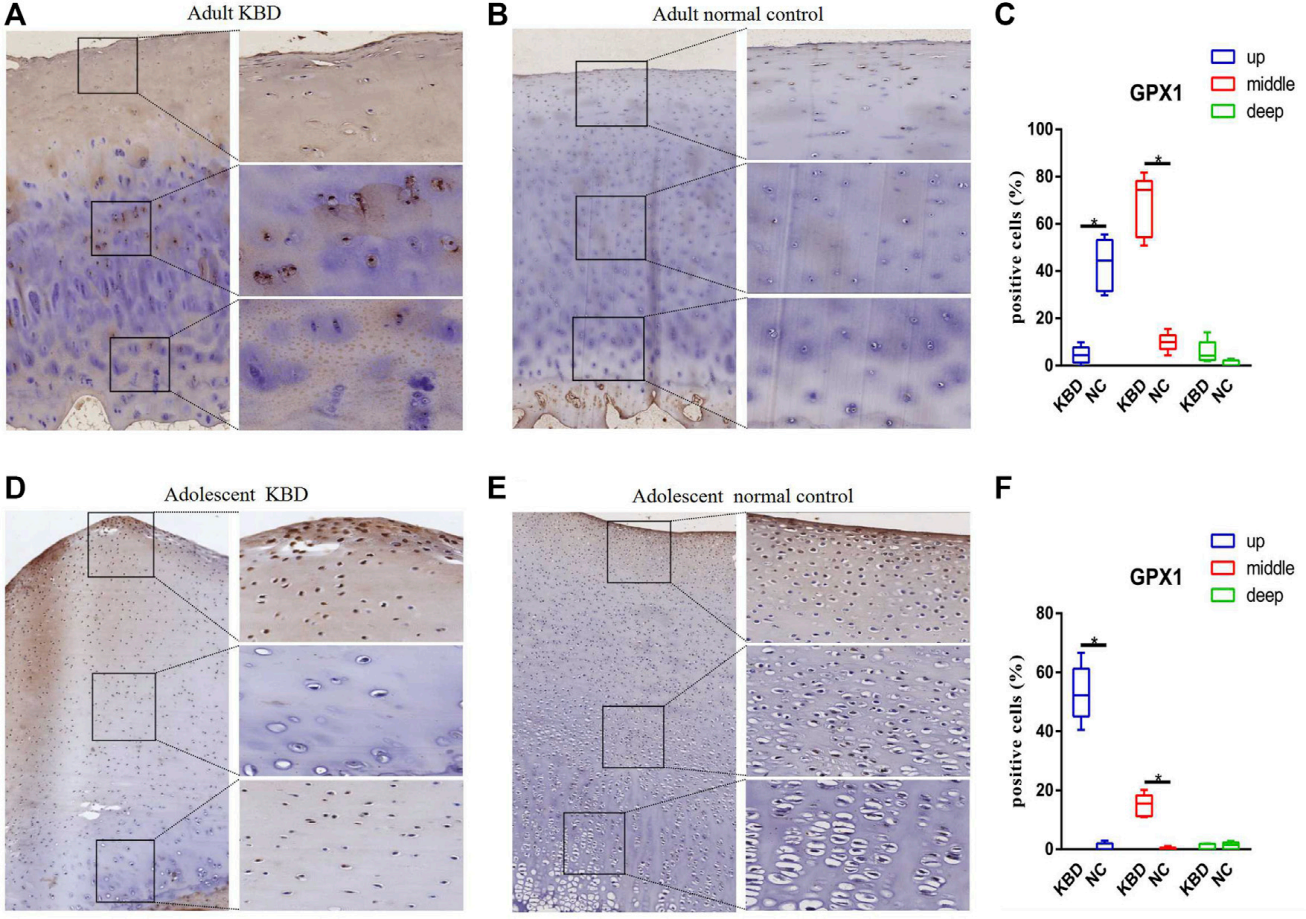
Representative immunohistochemistry staining of GPX1 in adult KBD **(A)**, adult normal control **(B)**, adolescent KBD **(D)**, and adolescent normal control **(E)** cartilage tissues (scale bar: left, 500 μm; right, 100 μm) and comparative quantification of positive cells of different areas (up, middle, and deep) in adult **(C)** and adolescent **(F)** cartilage tissues displayed by a box plot (n = 3). *p < 0.05.

**FIGURE 3:**
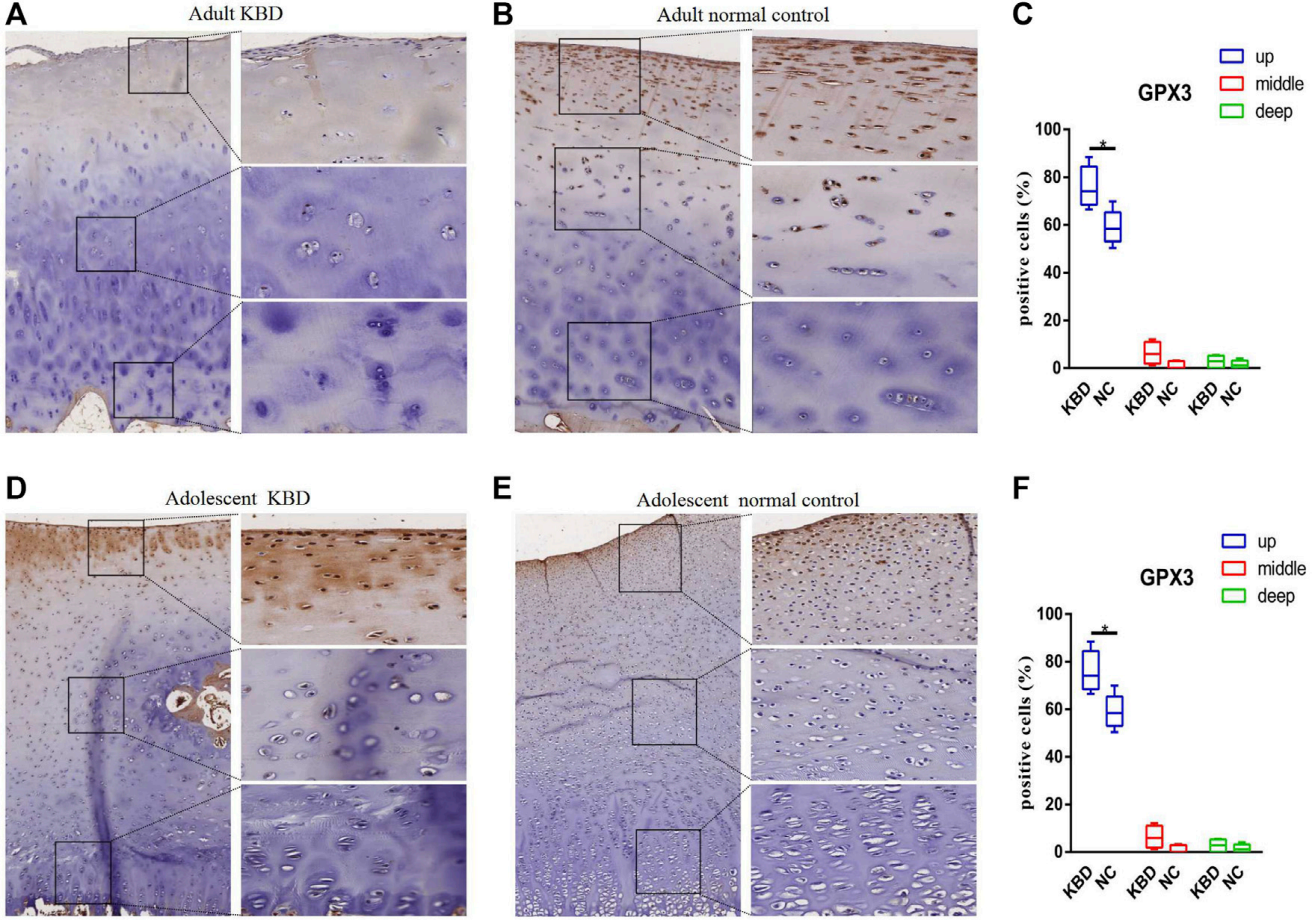
Representative immunohistochemistry staining of GPX3 in adult KBD **(A)**, adult normal control **(B)**, adolescent KBD **(D)**, and adolescent normal control **(E)** cartilage tissues (scale bar: left, 500 μm; right, 100 μm) and comparative quantification of positive cells of different areas (up, middle, and deep) in adult **(C)** and adolescent **(F)** cartilage tissues displayed by a box plot (*n* = 3). *p < 0.05.

**FIGURE 4:**
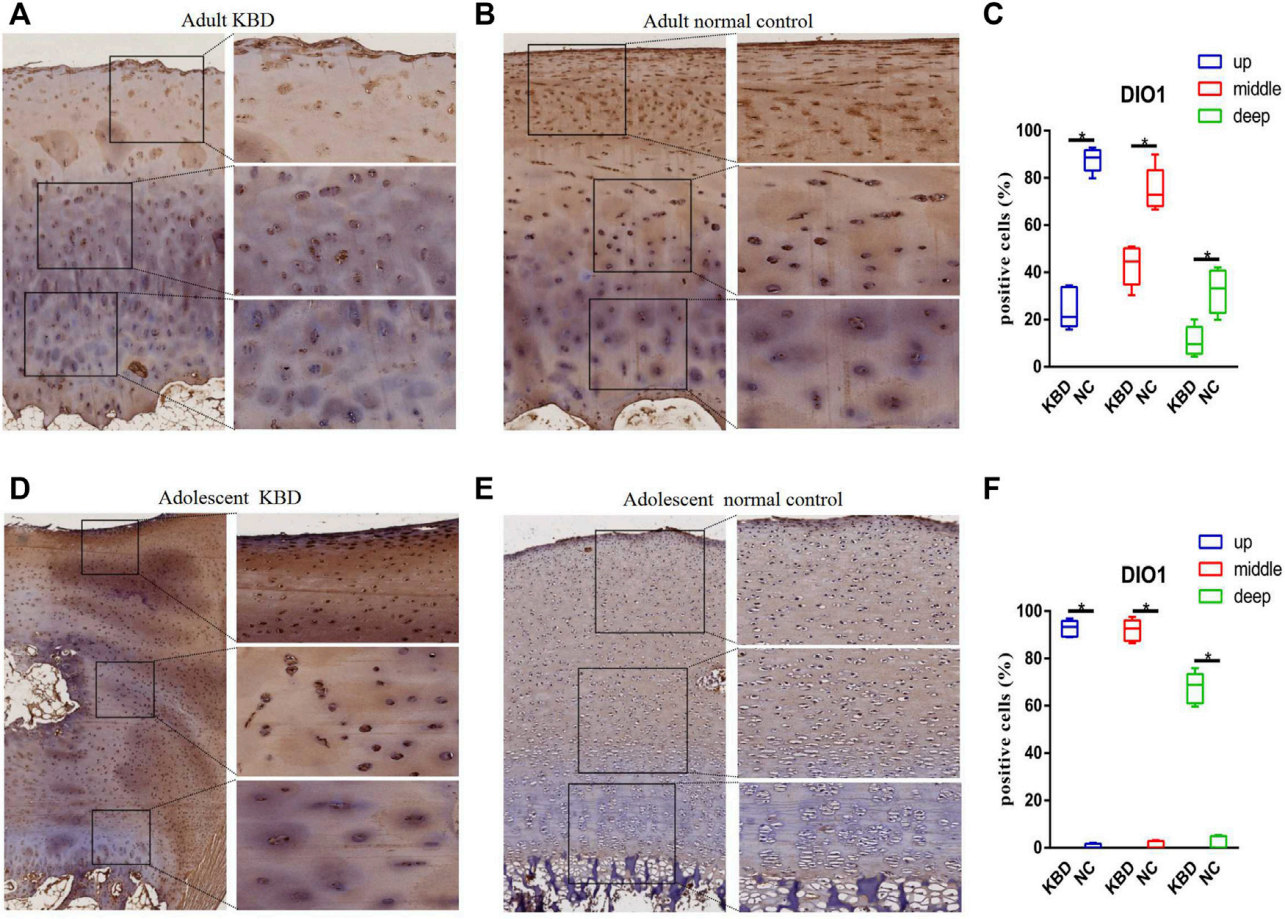
Representative immunohistochemistry staining of DIO1 in adult KBD **(A)**, adult normal control **(B)**, adolescent KBD **(D)**, and adolescent normal control **(E)** cartilage tissues (scale bar: left, 500 μm; right, 100 μm) and comparative quantification of positive cells of different areas (up, middle, and deep) in adult **(C)** and adolescent **(F)** cartilage tissues displayed by a box plot (*n* = 3). *p < 0.05.

**FIGURE 5:**
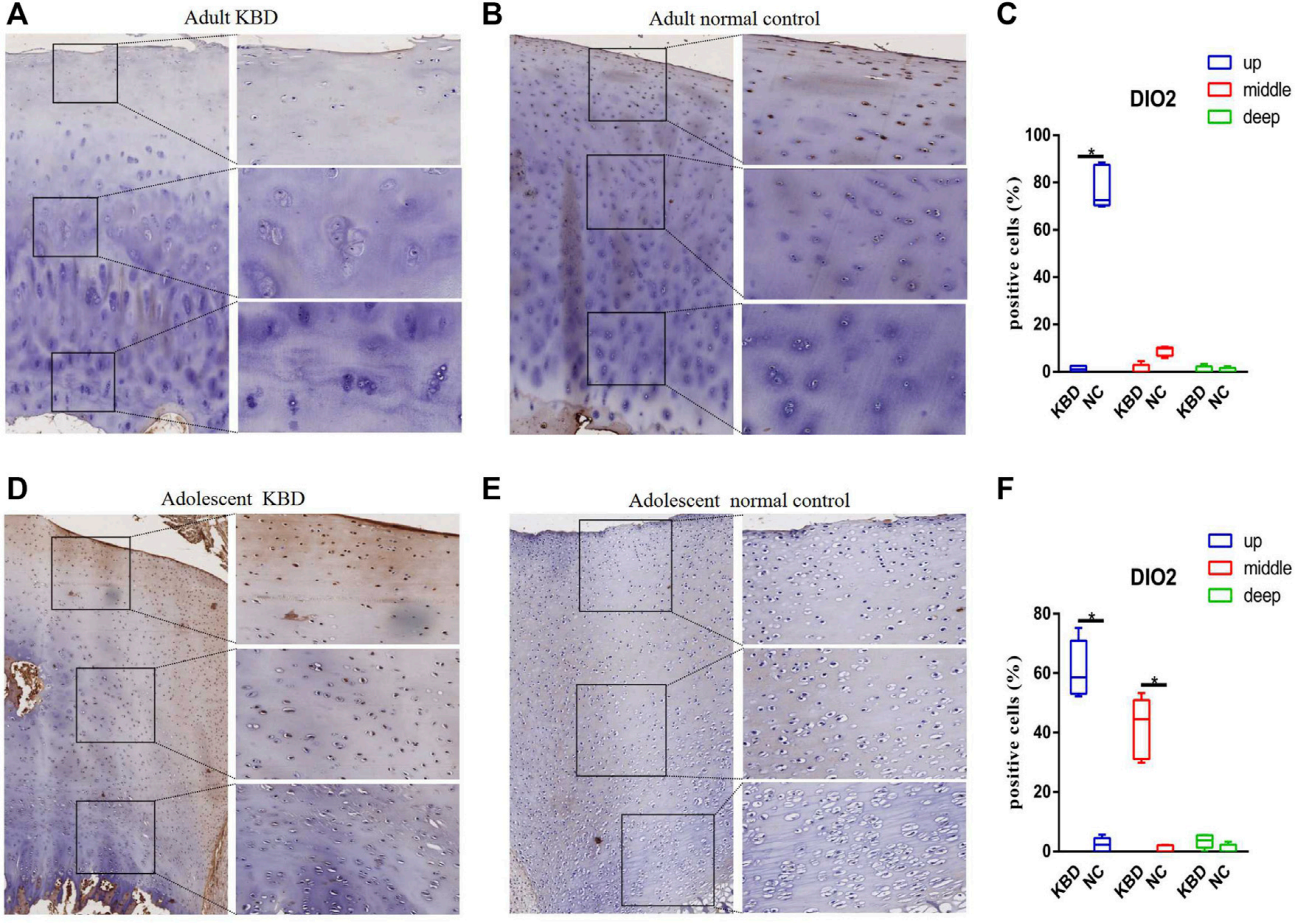
Representative immunohistochemistry staining of DIO2 in adult KBD **(A)**, adult normal control **(B)**, adolescent KBD **(D)**, and adolescent normal control **(E)** cartilage tissues (scale bar: left, 500 μm; right, 100 μm) and comparative quantification of positive cells of different areas (up, middle, and deep) in adult **(C)** and adolescent **(F)** cartilage tissues displayed by a box plot (*n* = 3). *p < 0.05.

**FIGURE 6:**
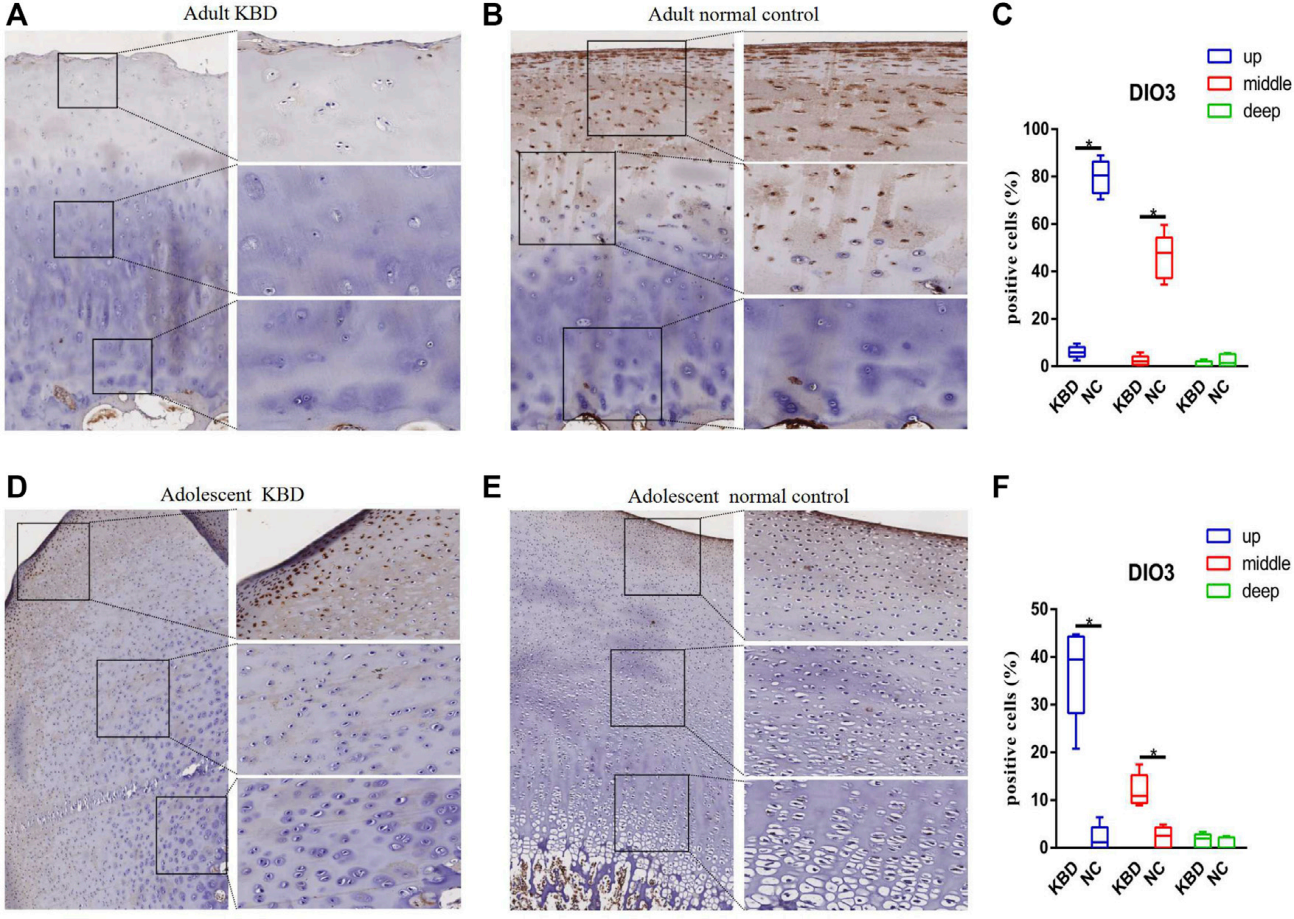
Representative immunohistochemistry staining of DIO3 in adult KBD **(A)**, adult normal control **(B)**, adolescent KBD **(D)**, and adolescent normal control **(E)** cartilage tissues (scale bar: left, 500 μm; right, 100 μm) and comparative quantification of positive cells of different areas (up, middle, and deep) in adult **(C)** and adolescent **(F)** cartilage tissues displayed by a box plot (*n* = 3). *p < 0.05.

The results demonstrated that the stronger positive staining for GPX1 was mainly in the cytoplasm in the middle zones of the articular cartilage in the adult and adolescent KBD groups compared with the normal subjects (Figure 2). In the same way, stronger immunopositive staining for GPX3 localized in the cytoplasm of the superficial zones was found in KBD cartilage (Figure 3). DIO-positive staining was observed on the cell membrane. For DIO1, the expression in KBD cartilage was reduced significantly compared to that in the normal subjects in the superficial, middle, and deep zones; in contrast, it was increased significantly in adolescent samples in all zones (Figure 4). For DIO2, the expression was reduced in the superficial zones in adult patients but was increased in the superficial and middle zones in adolescent patients (Figure 5). The expression of DIO3 was similarly reduced in the superficial and middle zones in adult patients but was increased in the superficial and middle zones in adolescent patients (Figure 6).

## Discussion

Dietary selenium, which is the core molecule in the pathway of oxidative stress inhibition and the regulation of endocrine physiology, is very important for the survival of mammals and plays a key role in neuronal function and male fertility (Burk and Hill, 2015). In humans, selenium is mostly taken up from the diet and then absorbed into the intestinal tract and transported to the liver, where it is mainly metabolized into selenocysteine (SEC). SEC is bound to selenoproteins and secreted into the plasma and peripheral tissues as a source of selenium (Ha et al., 2019).

Glutathione peroxidase is thought to use glutathione (GSH) as a cofactor to reduce hydroperoxides to corresponding alcohols (Stoytcheva and Berry, 2009). For instance, elevated levels of lipid hydroperoxide were found in GPX1 and GPX2 knockout mice, which suggests that GPX is essential for the prevention of the inflammatory response (Esworthy et al., 2001; Chu et al., 2004). Gastrointestinal GPX mainly exists in the epithelial inner wall of the gastrointestinal tract and was initially considered a barrier to prevent the absorption of hydrogen peroxide in the intestinal tract. The level of phospholipid hydroperoxide GPX is high in the testis and is indispensable for sperm maturation and embryogenesis (Kohrle, 2007; Kohrle, 2021). The activity of GPX in the whole blood of patients with KBD was lower, and there were significant differences in the frequency of GPX1 Pro198Leu genotypes and alleles between patients with KBD and controls (Xiong et al., 2010). In the Tibetan population, haplotype analysis of SNPs rs1050450, rs1800668, and rs3811699 in the GPX1 gene showed a significant correlation with KBD (Huang et al., 2013). GPX3 CpG showed hypermethylation in KBD patients, which decreased the antioxidant function of GPX3 and had a positive effect on chondrocyte apoptosis (Han et al., 2018; Zhang et al., 2022). In this study, GPX1 and GPX3 were found to have a differential transcriptional level in KBD chondrocytes compared with normal controls. The mRNA levels of GPX1 and GPX3 were also upregulated in KBD articular cartilage in both children and adults, which could be involved in excessive oxidative stress in KBD patients (Wang et al., 2013; Dai et al., 2016). In addition, Yan et al. suggested that knocking down GPX1 in ADTC5 cells could cause oxidative stress characterized by increased ROS levels, which could lead to the inhibition of chondrocyte proliferation (Yang et al., 2017). Bone marrow stromal cells cultured in a Se-deficient medium showed ROS accumulation and decreased expression of GPX and thioredoxin reductase, resulting in micronucleus formation, which is an indicator of chromosome damage (Ebert et al., 2006). ROS can inhibit mitochondrial oxidative phosphorylation and ATP production, thus destroying the balance between ECM catabolism and anabolism (Johnson et al., 2000; Henrotin et al., 2003), and antioxidant treatment can eliminate this effect. In fact, many attempts have been made to treat OA by targeting regulators involved in cartilage oxidative stress (Ebert et al., 2006; Loeser, 2009).

Iodine is an indispensable trace mineral for the synthesis of thyroid hormones, and iodine deficiency may affect the growth and development of bones (Rohner et al., 2014). Iodine deficiency usually coexists with selenium deficiency in KBD endemic areas, which is considered to be a potential risk factor for KBD (Yao et al., 2011; Yang et al., 2016). Similarly, several studies have shown different iodine levels in the blood and urine of KBD patients compared with normal controls (Moreno-Reyes et al., 1998; Moreno-Reyes et al., 2001; Shi et al., 2011). DIO1 and DIO2 can remove an iodine atom from the casein outer ring of tetraiodothyronine (T4) to produce active triiodothyronine (T3). However, DIO3 only catalyzes the deiodization of the inner ring of T4, resulting in the formation of the inactive product RT3. Deiodinases participate in the regulation of the dynamic balance, development, growth, and metabolism of thyroid hormones on the basis of cell specificity by affecting the level of intracellular T3 (Bianco and da Conceicao, 2018). In our study, DIO1, DIO2, and DIO3 transcript levels increased, but their protein levels, as indicated by immunohistochemistry, decreased in adults with KBD. Similarly, previous studies have shown that transcript levels do not necessarily reflect the functional activity of deiodinases, which might be related to posttranscriptional modification or inactivation, but the specific mechanism is unclear (Bianco and Kim, 2006; Kohrle, 2021). Both H. Nagase and N. Bomer collected cartilage samples from patients with OA, and overexpression of DIO2 was observed, which may increase the degradation of cartilage and eventually lead to the pathogenesis of OA (Nagase et al., 2013; Bomer et al., 2015). However, Cheng transfected human articular chondrocytes with a specific siRNA to significantly decrease the mRNA expression of DIO2, GPX1, and TR1, and the results showed that inhibition of DIO2 significantly increased IL-1β gene expression, indicating that DIO2 had an anti-inflammatory effect on the body (Cheng et al., 2012). Although there is little literature on the role of DIO1 in cartilage development, Li et al. (2020) found that, compared with normal subjects, the methylation frequency of the DIO3 gene promoter is significantly higher in KBD patients, and the hypermethylation of DIO3 might increase the risk of KBD by more than 4 times. This evidence suggests that the expression of DIOs could play a crucial role in the pathogenesis of KBD.

## Conclusion

Se deficiency has been considered a major environmental risk factor for KBD for a few decades. A number of studies have been performed to verify the causal relationship between selenium and KBD, but there is less research that directly focuses on the mRNA expression of selenoproteins in KBD chondrocytes. Therefore, in this study, the mRNA expression levels of 25 selenoproteins in KBD chondrocytes were detected, and IHC was performed to detect the protein expression levels of the selenoprotein genes with differential mRNA expression in cartilage tissue sections, which may contribute to the mechanism of selenoprotein involvement in KBD. In this study, we observed the differential expression of GPX1, GPX3, DIO1, DIO2, DIO3, and SELENOP in KBD cartilage, which indicated that selenoproteins were metabolically disordered in KBD patients and suggested that selenoproteins could play a crucial role in the pathogenesis of KBD.

## Data Availability

The original contributions presented in the study are included in the article/Supplementary Material; further inquiries can be directed to the corresponding authors.
