# Supplementary material for: Detection of selenoprotein transcriptome in chondrocytes of patients with Kashin–Beck disease
Source: Front Cell Dev Biol. 2023 Feb 17;11:1083904. doi: 10.3389/fcell.2023.1083904 (PMC9981956; doi:10.3389/fcell.2023.1083904)
Supplement: Supplementary file 1 [file Table1.pdf]

Table S1 Characteristics of adolescent/adult patients with KBD and control used for and IHC

Sample pair	KBD		Normal	
	Age (years)	Gender	Age(years)	Gender
Adult				
1	58	Female	55	Female
2	64	Female	60	Female
3	53	Male	51	Male
4	53	Female	59	Female
5	54	Male	55	Male
6	55	Male	49	Male
Adolescent				
1	7	Male	6	Male
2	9	Male	7	Male
3	8	Male	8	Male
4	6	Female	5	Female
-	-	-	9	Male
-	-	-	7	Female
Mean	7.5	-	7	-
