# Supplementary material for: Detection of selenoprotein transcriptome in chondrocytes of patients with Kashin–Beck disease
Source: Front Cell Dev Biol. 2023 Feb 17;11:1083904. doi: 10.3389/fcell.2023.1083904 (PMC9981956; doi:10.3389/fcell.2023.1083904)
Supplement: Supplementary file 2 [file Table2.pdf]

Table S2 Real-time PCR primers used in this study

Target gene	Forwardprimer(5'→3')	Reverseprimer(5'→3')	Amplicon
GAPDH	TGTTGCCATCAATGACCCCTT	CTCCACGACGTACTCAGCG	202
GPX1	TCTGGCTACTCTCTCGTTTCCT	ATGAGTCACCGGGATTTTGC	98
GPX3	AGAAGTCGAAGATGGACTGCC	AGAGGACGTATTTGCCAGCAT	120
GPX4	AGCAAGATCTGCGTGAACGG	GGTGAAGTTCCACTTGATGGC	108
DIO1	GGCGAGAAGACGGGTATGAC	ACGGACCTTCAAGACGAACC	102
DIO2	ACAAACAGGTGAAATTGGGTGA	ACAAACAGGTGAAATTGGGTGA	146
DIO3	TGACACTGATGTGCTGAGCC	GATTTCCAGTGAGCCAGGCA	131
SELENOF	TCGAAAGCACCCAGTGATTGT	TCCACTCCAGAGCCTGATCC	135
SELENOH	GGACTGGGATTAAGAAGGGGC	GGACACAAAGCCAAATCTCCC	109
SELENOI	TTCTCCATGCTTGCTGTTCA	GGCAAAAGCTGTTCCAACCA	112
SELENOK	CGTCTCTTTCAGCAGCCAATG	GCCCCTGTCGGTTTCTGTAT	120
SELENOM	CTTTCGTCACGCAGGACATTC	TCTCTTCGCGGGTCATTTCA	141
SELENON	GGTTCTCCCCTGCTCAGTTC	CCCGTAAAGCCACTCCATGT	125
SELENOO	GCAGAGAAATGCTGCCTTCTT	TCATGTTGTCGGTGTTGAGC	110
SELENOP	TATGTAAGCAACCCCCAGCC	GCACAGGTATCAGCTGGCTT	107
SELENOS	CTGGCGGATGAGGCTAAGAA	CACTGTGAAAAGCGTGCGTA	101
SELENOT	TCTCTGTTTGCCTTGGGTTGA	AAGAGATGAGCGTCAACAACAC	100
SELENOW	CCGAGGCTCCATCCTTTCTG	CAATAAACGACTCGGACGGC	148
TXNRD1	ATGTCATGTGAGGACGGTCG	TCTGCCCTCCTGATAAGCCT	138
TXNRD2	TACTAGCCCCGACACTCAGA	TATGGGTGTCAGCTCAGGC	112

TXNRD3	GGGGCAGGCATTATGTGACT	AGAGACAACCTGTAGCCCCA	132
SEPHS2	AGTACGGAGAGGGTCACCAA	CCACGAGGCAGGACTTCAAT	106
