# Supplementary figures and images for: Detection of selenoprotein transcriptome in chondrocytes of patients with Kashin–Beck disease
Source: Front Cell Dev Biol. 2023 Feb 17;11:1083904. doi: 10.3389/fcell.2023.1083904 (PMC9981956; doi:10.3389/fcell.2023.1083904)

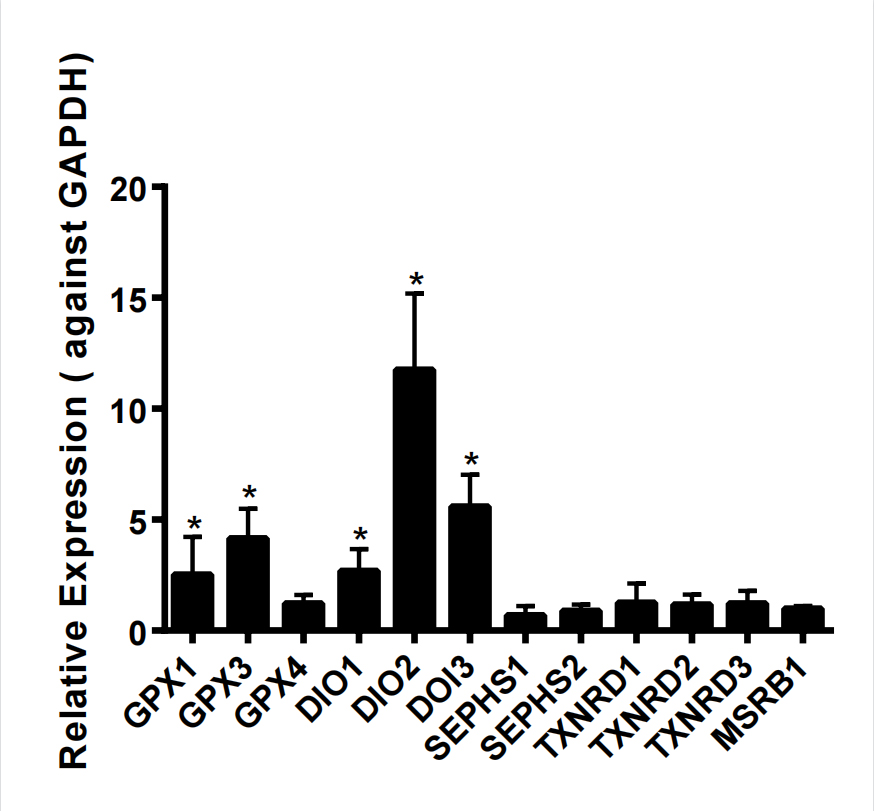

Supplement: Supplementary file 3 [file Image1.JPEG]

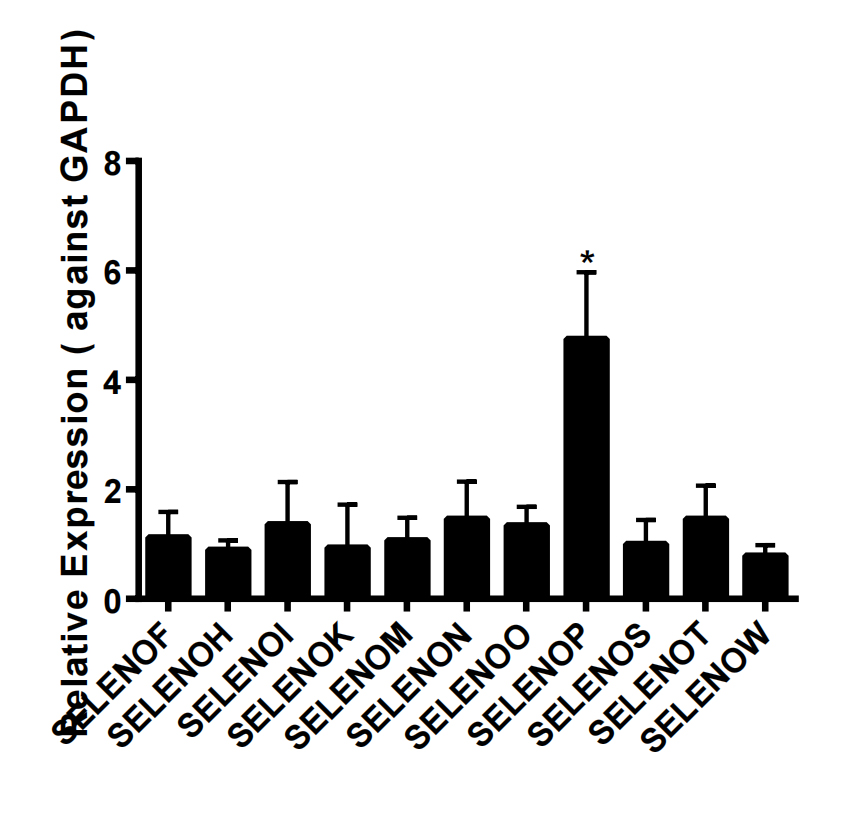

Supplement: Supplementary file 4 [file Image2.JPEG]
